# Counting circulating endothelial cells in allo-HSCT: an ad hoc designed polychromatic flowcytometry-based panel versus the CellSearch System

**DOI:** 10.1038/s41598-018-36442-9

**Published:** 2019-01-14

**Authors:** Camillo Almici, Arabella Neva, Cristina Skert, Benedetto Bruno, Rosanna Verardi, Andrea Di Palma, Andrea Bianchetti, Simona Braga, Giovanna Piovani, Valeria Cancelli, Paola Omedè, Kurt Baeten, Gianluca Rotta, Domenico Russo, Mirella Marini

**Affiliations:** 1 Laboratory for Stem Cells Manipulation and Cryopreservation, Dpt of Transfusion Medicine, ASST Spedali Civili, Brescia, Italy; 2 Chair of Hematology, Unit of Blood Diseases and Stem Cell Transplantation, University of Brescia, ASST Spedali Civili, Brescia, Italy; 3 BMT Unit, Department of Molecular Biotechnology and Health Sciences, University of Torino, A.O.U Città della Salute e della Scienza di Torino, Torino, Italy; 4 Biology and Genetics Division, Department of Molecular and Translational Medicine, University of Brescia, Brescia, Italy; 5 Global Scientific and Medical Affairs, Janssen Diagnostics, Beerse, Belgium; 6 Scientific Affairs, BD Biosciences Italia, Milano, Italy

## Abstract

Physio-pathologic interrelationships between endothelial layer and graft-versus-host disease (GVHD) have been described leading to assess the entity “endothelial GVHD” as the early step for clinical manifestations of acute GVHD. The availability of the CellSearch system has allowed us to monitor Circulating Endothelial Cells (CEC) changes in allogeneic hematopoietic stem cell transplantation (allo-HSCT) as useful tool to help clinicians in GVHD diagnostic definition. We have compared CEC counts generated by an *ad hoc* designed polychromatic-flowcytometry (PFC) Lyotube with those of the CellSearch system. CEC were counted in parallel at 5 timepoints in 50 patients with malignant hematologic disorders undergoing allo-HSCT (*ClinicalTrials.gov*, NCT02064972). Spearman rank correlation showed significant association between CEC values at all time points (p = 0.0001). The limits of agreement was demonstrated by Bland Altman plot analysis, showing bias not significant at T1, T3, T4, while at T2 and T5 resulted not estimable. Moreover, Passing Bablok regression analysis showed not significant differences between BD Lyotube and CellSearch system. We show that CEC counts, generated with either the CellSearch system or the PFC-based panel, have a superimposable kinetic in allo-HSCT patients and that both counting procedures hold the potential to enter clinical routine as a suitable tool to assist clinicians in GVHD diagnosis.

## Introduction

Allogeneic hematopoietic stem cell transplantation (allo-HSCT) has strongly improved, over the past decades, the cure rate of several onco-hematologic diseases^1^. However, despite of these promising results, graft-versus-host disease (GVHD) remains one of the major factors influencing patients survival^2,3^. In fact, GVHD overall incidence ranges between 30% and 60% accounting for a 50% mortality rate, particularly in the more severe presentation^4^. Nowadays, GVHD diagnosis in routine clinics is still based on patient symptoms, clinicians expertise and affected tissues histology^5^. In order to reduce the need of invasive procedures, clinical researchers are hardly engaged in seeking specific and objective biomarkers in peripheral blood (PB) for improving GVHD diagnostic definition^6^. The recent definition of the physio-pathologic interrelationship between endothelium and GVHD has confirmed that vascular endothelium is an early target for donor T-lymphocytes^7,8^. Currently, circulating endothelial cells (CEC) are considered a specific and sensitive marker of endothelial damage in a variety of pathological conditions^9–14^. However, due to their rareness and complex phenotypes, no consensus on CEC identification and count has been so far fully obtained^15–17^. Therefore, the fine tuning of a standardized approach for CEC identification and count could result crucial in order to move their monitoring into clinical practice. We recently joined a multicenter study (S.C.EN.I.C. Network: Standardization of Circulating ENdothelIal Cells) that, utilizing a highly optimized method based on a polychromatic flowcytometry (PFC) Lyotube, proved the possibility to reach a high level standardization in CEC count and analysis^18,19^. Moreover, a physiological baseline range of CEC values has been provided for healthy subjects, becoming a valid reference in endothelial dysfunctions^19^. Today, CellSearch system represents the only commercially available procedure that guarantees standardization in CEC identification and count with high-level reproducibility, specificity and sensitivity^10,13,14,20^. We present the results of a comparative study of CEC counts (5 different timepoints) performed in parallel by means of an *ad hoc* designed PFC Lyotube and the CellSearch system in a cohort of 50 patients with malignant hematologic diseases undergoing allo-HSCT (*ClinicalTrials.gov*, NCT02064972). Although preliminary, our results can be the basis for future large-scale confirmatory study.

## Material and Methods

### Patients

Between June 2014 and October 2015, we prospectively analyzed 50 patients with malignant hematological disorders undergoing allo-HSCT. CEC counts were performed in parallel by means of two different methodologies: (1) polychromatic flow cytometry (PFC) using, all along the study, a single batch of pre-formatted lyophilized-reagent tubes (Becton Dickinson, San Jose, CA, USA; Lyotube, Custom cat # 623920)^18,19^; (2) CellSearch system (Janssen Diagnostics LLC, Raritan, NJ, USA). The local research and ethics committee (Comitato Etico della Provincia di Brescia, document NP 1574 of the 14^th^ January 2014 and Comitato Etico interaziendale AOU Città della Salute e della Scienza di Torino, document 0037975 of the 10^th^ April 2014) approved the study protocol and all patients and controls provided written informed consent, in accordance with the Declaration of Helsinki. The trial was registered on *ClinicalTrials.gov* (NCT02064972).

CEC counting was scheduled before and after conditioning regimen, at time of hematopoietic engraftment, at day +28 in the absence of GVHD, at time of GVHD onset and 1 week after steroid treatment. Day +28 was selected as a CEC counting timepoint in patients without GVHD, since the median time of GVHD onset in our previous series was 27 days^14^. Therefore, CEC values at day +28 in patients without GVHD were compared with CEC values at GVHD onset. Thus, CEC count during study was performed at the following five time points: T1 (pre-conditioning), T2 (pre-transplant), T3 (engraftment), T4 (GVHD onset or day +28), T5 (1 week after steroids).

During study period, levofloxacin prophylaxis was continued until neutrophil recovery, and fluconazole/itraconazole until immunosuppressive drugs has been suspended, while trimethoprim-sulphamethoxazole was used for *Pneumocystis jirovecii* prevention. Cytomegalovirus was weekly PCR monitored, and patients testing positive have received ganciclovir/foscarnet treatment. Fungal infections have been diagnosed according to published revised criteria^21^. GVHD diagnosis and grading were defined according to commonly accepted criteria^22^.

### Controls

Healthy volunteers (age 18 to 65 years) with normal blood parameters and pressure values served as controls. As previously reported^19^, healthy subjects were excluded if presenting at least one of the following parameters out of normality ranges^12^: blood pressure, glycaemia, cholesterol value. Smokers, healthy women within two weeks from menstrual period, individuals fasted within 12 hours, subjects with endometriosis, with active duodenal or gastric ulcer, HIV, HBV or HCV positive, subjects that received drug treatments in the preceding 48 hours or with present or previous neoplastic, infectious, inflammatory or cardiovascular diseases were also excluded. CEC counts were performed with CellSearch (n = 17) and with PFC (n = 21).

### Blood specimen collection

PB samples have been drawn from central catheter, in order to decrease risks of endothelial cell detachment due to traumatic damage from venipuncture. Samples for CellSearch count were collected in specifically dedicated tubes (CellSave Preservative Tubes, Janssen Diagnostics LLC, Raritan, NJ, USA), that guarantee the reproducibility of results up to 96 hours from blood drawn; while samples for PFC count were collected in three EDTA (2 mg/ml) tubes (BD K2E EDTA, Becton Dickinson Biosciences - BD, San Jose, CA, USA). Leukocyte count, determined on each first drawn tube, was used for double platform calculation.

### Polychromatic flowcytometry (PFC)

CEC determinations were performed within 4 h from collection^18,19^, with the purpose to avoid any detrimental effects on counting performance over time, as reported for both CEC^19^ and EPC^23^. As previously described^18,19^, PB volume containing 20 × 10^6^ leukocytes underwent erythrocyte-lysis with 45 ml of Pharm Lyse solution (BD Biosciences), followed by centrifugation (400 g, 10 min, room temperature) and wash with 2 ml of Stain Buffer containing bovine serum albumin (BD Biosciences). Surface staining was accomplished by adding the resuspended pellet of each sample to the Circulating Endothelial Cell Lyotube kit (Becton Dickinson, Custom cat #623920) (Panel tube: CD146PE, CD34PE-Cy7, CD309AlexaFluor647, CD45APC-H7, 7AAD; Control tube: IsotypePE, CD34PE-Cy7, IsotypeAlexaFluor647, CD45APC-H7, 7AAD) and 1 µM Syto-16 (Thermo Fisher Scientific, Eisai, Medipost - US) was added as liquid drop-in. Samples incubated in the dark for 30 min at 4 °C were then washed (2 ml of Stain Buffer with BSA, BD Biosciences) and re-suspended in 1.5 mL of FACSFlow (BD Biosciences). Finally, 2–4 × 10^6^ events/sample with lympho-monocyte morphology were acquired by flow cytometry (FACSCanto II, BD Biosciences). A threshold combination was set on FSC and FITC channel (Syto16) to exclude very small and non-nucleated events. Data were analyzed using FACSDiva v 6.1.3 (BD), and FACSuite v1.05 (BD Biosciences) and FlowJo v 8.8.6 (TreeStar, Ashland, OR) software.

CEC were defined as 7-AAD^neg^/syto16^pos^/CD45^neg^/CD34^bright^/CD146^pos^ and counted by a dual-platform method applying the following formula^18^:

$$CEC/mL=\frac{\#CE{C}_{panel}-\#CE{C}_{control}\times \#Total\,CD{34}_{panel}/\#Total\,CD{34}_{control}}{\#Lymphocyt{e}_{panel}}\times Lymphocyte\,Count\times 1000$$

### CellSearch System

CEC counts were performed, within 48 hours from collection, using the Circulating Endothelial Cell isolation kit (research use only) on the CellSearch system (Janssen Diagnostics LLC, Raritan, NJ, USA), allowing to standardize the process of sample collection, cellular selection, monoclonal antibodies labelling, analysis and enumeration of CEC^10^. CEC were defined as CD146^+^/CD105^+^/DAPI^+^/CD45^−^ cells and their values expressed per mL of PB.

### Statistical Analysis

Standard descriptive statistics were used to summarize the patient sample. Continuous data were expressed as mean ± standard deviation and as median (range). Mann-Whitney U test was used in univariate analysis for comparison of continuous variables, and chi-squared test for comparison of categorical variables. Spearman’s rank correlation coefficient was calculated to estimate the association between PFC and CellSearch generated CEC values. The comparability between the two methods of CEC count (PFC Lyotube and CellSearch) was assessed by Passing Bablok regression analysis and by Bland Altman plot analysis. Passing Bablok regression analysis was used to detect constant or proportional differences between the two methods. In Bland Altman analysis, the mean of the differences between the paired measurements of the two methods (bias) and the limits of agreement were estimated. The limits of agreement defined the range within which 95% of the differences were included. The bias was not significant if the 95% confidence interval of the mean difference included the value 0 (line of equality on the Bland Altman plot).

For each method, a multivariate logistic regression analysis was performed at each time-point, to assess the correlation between GVHD and CEC values in the presence of possible interfering factors (i.e. patient and transplant-related variables, infectious events). The count of CEC/ml of PB and the relative increase of CEC values at each time point (T) were included in all analyses. All p values were 2-sided and p < 0.05 was considered statistically significant.

## Results

### Allografting

We enrolled 50 patients with median age 51.5 years (range 18–69; 30 males and 20 females) undergoing allo-HSCT from either HLA-matched familial (n = 8; 16%), unrelated (n = 26; 52%) or haploidentical donor (n = 16; 32%) for their malignant hematologic disorders (24 AML, 10 ALL, 4 HD, 3 NHL, 1 CLL, 5 MDS, 3 Chronic Myeloproliferative Disorders). At time of enrollment 29 patients (58%) were in complete response (CR), 19 patients (38%) in partial response/CR > 1 and 2 patients (4%) in progression from their diseases. Thirty-nine (78%) patients received hematopoietic stem cells from mobilized peripheral blood and 11 (22%) from bone marrow. The conditioning regimen was marrow-ablative in 30 patients (60%) and reduced intensity in 20 patients (40%). Engraftment was reached in 48 patients at a median time of 22 days (range 14–40); two patients died in aplasia, 1 from infection and 1 for toxicity (Table 1). No clinical and transplant differences were recorded between patients developing GVHD and those without GVHD, except for acute leukemia diagnosis (p = 0.03), haploidentical donor (p = 0.02) and F/Bu2 reduced conditioning (p = 0.03) (Supplemental Table S1). 20/50 patients (40%) presented GVHD at a median of 23 days (range 14–113) post-transplant. GVHD was grade I in 3/20 (15%), grade II in 16/20 (80%), grade III in 1/20 patients (5%), grade IV in 0/20 patients (0%), respectively. In 12/20 patients (60%) GVHD presented skin involvement, and in 10/20 patients (50%) gut involvement.

**Table 1:** Patients’ and transplant’s characteristics (n = 50).

Characteristics	Values	%
Age (years), median (range)	51.5 (18–69)	
Sex		
Male	30	60
Female	20	40
Diagnosis		
Acute Leukemias	34	68
Lymphomas/CLL	8	16
MDS	5	10
CMS	3	6
Disease status		
CR	29	58
PR/CR >1	19	38
Progression	2	4
Donor		
MUD	26	52
MRD	8	16
Haploid	16	32
HPC source		
MPB	39	78
BM	11	22
Conditioning regimen		
MAC	30	60
RIC	20	40
MA conditioning		
BU/CY	11	22
FBu4	4	8
TBI/CY	4	8
TBI/F/Th	3	6
TBF	8	16
RIC conditioning		
Th/CY/F	2	4
TBI/F/Th(r)	2	4
FBu2	6	12
Th/CY	5	10
TBF(r)	4	8
Th/F	1	2
GVHD prophylaxis*		
CyA/MTX	33	66
CyA/MMF	17	34
ATG	19	38
Engraftment	48	100 (48 evaluable patients)
Time of engraftment (days), median (range)	22 (14–40)	

CLL: Chronic Lymphocitic Leukaemia, MDS: Myelodiplastic Syndrome, CMS: Chronic Myeloproliferative Syndromes, CR: complete remission, PR: partial remission, MUD: Matched Unrelated Donor, MRD: Matched Related Donor, Haploid: haploidentical related donor, MPB: Mobilised Peripheral Blood, BM: Bone Marrow, MAC: myeloablative conditinong, RIC: reduced intensity conditioning, BU: Busulphan, CY: Cyclophosphamide, F: Fludarabine, TBI: Total Body Irradiation, Th: Thiotepa, TBF: Th/BU/F, r: RIC, CyA: Cyclosporin A, MTX: Methotrexate, MMF: Mofetil Micofenolate, ATG: Anti-Lymphocyte Globulin.

*In haploidentical transplantation GVHD prophylaxis included CyA/MMF and Cyclophosphamide post stem cell reinfusion (100 mg/kg total dose: day +3 and +5).

### CEC counting at baseline

The median CEC/ml in patients at T1 (pre-conditioning) was 24 (range 3–175) with PFC and 24 (range 2–786) with CellSearch (p = 0.63) in comparison to a value in healthy subjects of 13 (range 2–57) (p = 0.005) and 2 (range 1–14) (p = 0.0001), respectively (Supplemental Fig. S1).

### CEC counting with polychromatic flow-cytometry

**Figure 1:**
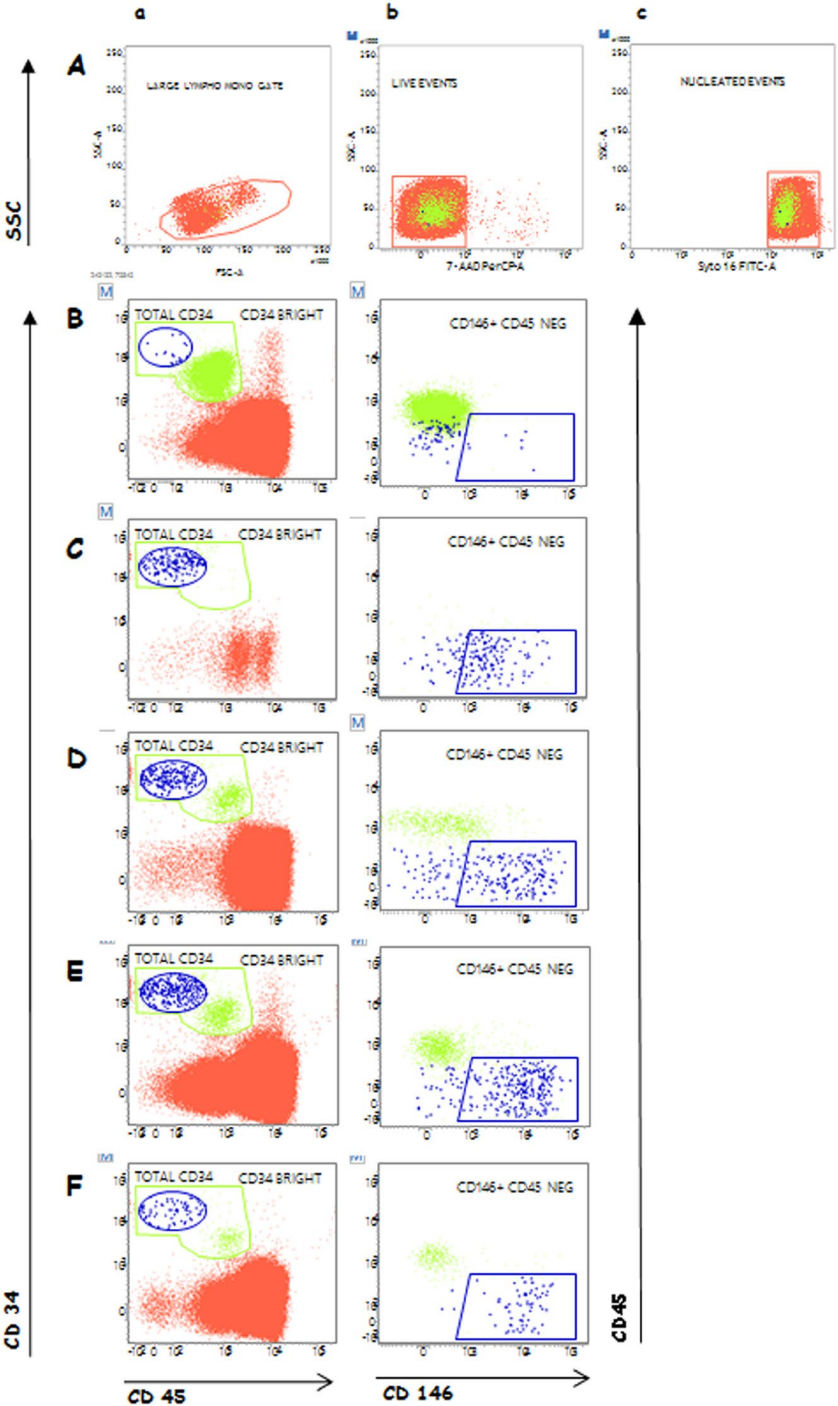
Flow cytometry identification and count of CEC in PB samples of a representative patient. CEC were analyzed and counted in an AML patient (57 years), conditioned with Fludarabine/Bu4 regimen and receiving HPC-A from a matched unrelated donor (engraftment at day +28, skin GVHD diagnosed at day +45). Panel A: (a) Events showing lympho-monocyte morphology were gated in a FSC/SSC plot. (b) Dead cells were excluded because 7-AAD positive and afterwards (c) nucleated events were gated. Cells resulting from the logical combination of the three gates intersection were analyzed for CD45 and CD34 expression. Panel B–F: Two subpopulations, showing different levels of CD34 surface expression, were separately gated: CD34 positive cells, being CD45dim, represent the hematopoietic stem cell compartment (green dots), and CD34 bright cells, resulting CD45 negative (blue dots). Both subpopulations were than analyzed for CD146 expression (right column): CEC resulted CD34 bright/CD45 negative/CD146 positive (blue dots). The plot analysis are shown at the different timepoints during allo-HSCT (panel B: T1 pre-conditioning; panel C: T2 pre-Transplant; panel D: T3 engraftment; panel E: T4 GVHD onset; panel F: T5 1 week after steroids). The corresponding results of CEC calculations are shown in Supplemental Table S2.

CEC counting in a representative patient at the five different timepoints is presented in Fig. 1. For a satisfactory identification and counting of CEC, a strategy based on a logical combination of gates is first applied on events displaying lympho-monocyte morphology, being alive (7-AAD neg) and nucleated (syto-16 pos) (Fig. 1, panel A, a–c). Afterward, cells being bright for CD34, negative for CD45 and positive for the endothelial cell marker CD146 (Fig. 1, panel B–F) are counted CEC. The corresponding raw data for calculating CEC values are reported in Supplemental Table S2.

**Figure 2:**
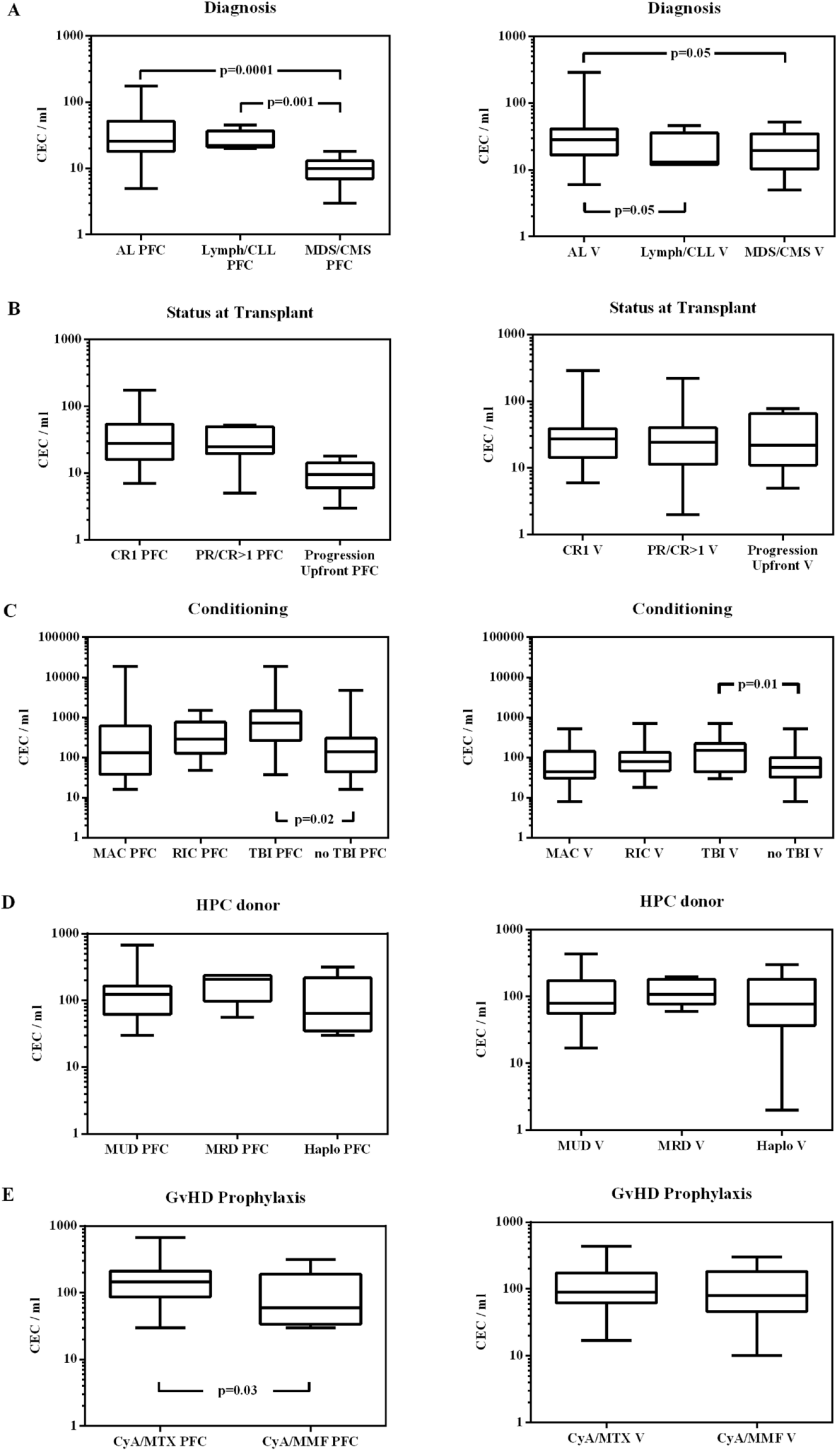
CEC counts during allogeneic hematopoietic stem cell transplantation. CEC counts performed with CellSearch (V) and with polychromatic flow cytometry (PFC) are shown on right and left column, respectively. CEC values at T1 (pre-conditioning) are shown in row (**A**) according to diagnosis, in row (**B**) to disease status, while CEC values at T2 (pre-transplant) are shown in row (**C**) according to conditioning, in row (**D**) to HPC donor, and CEC values at T3 (engraftment) are shown in row (**E**) according to GVHD prophylaxis.

**Figure 3:**
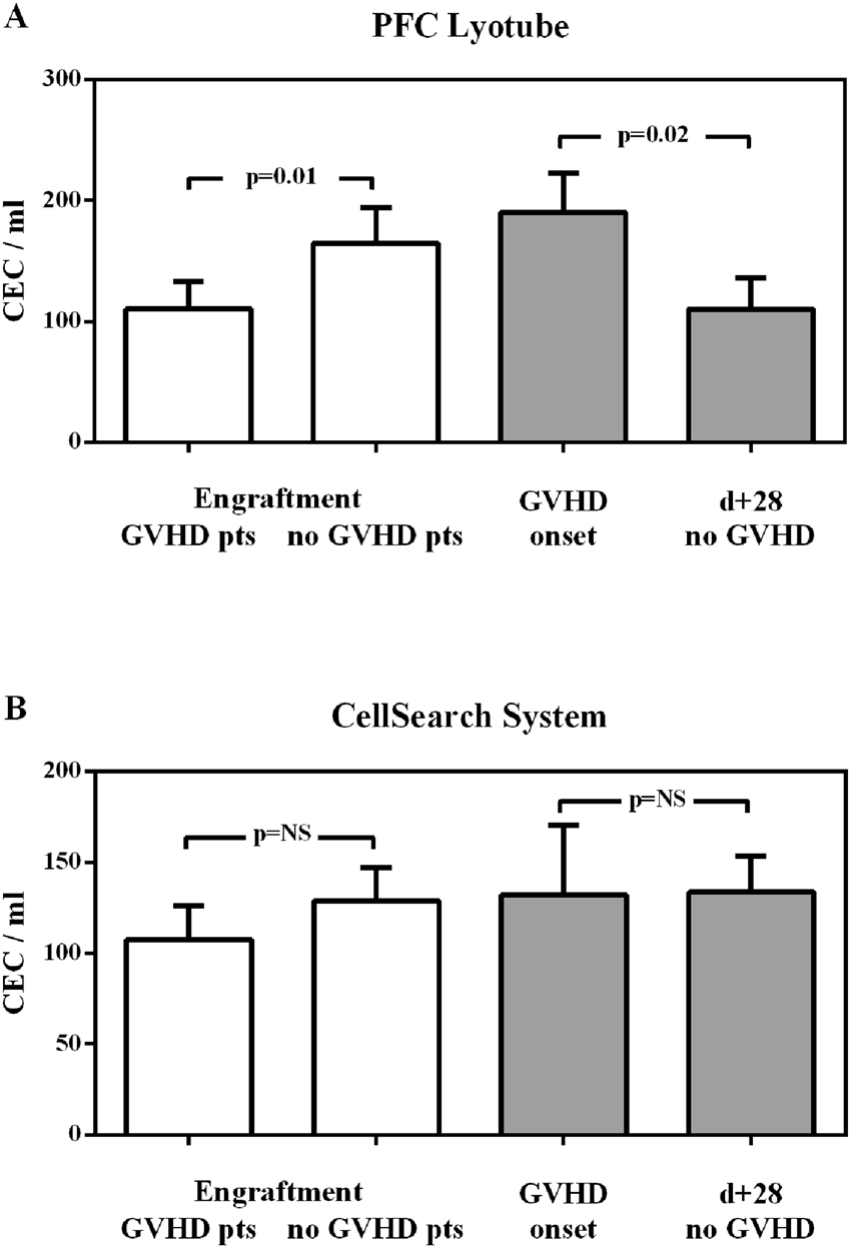
CEC counts in patients with and without GVHD. CEC counts performed with PFC Lyotube (panel A) and with CellSearch System (panel B) at T3 (engraftment) and T4 (GVHD onset or day +28) timepoints.

In PFC-generated CEC values, no differences were detected between patients developing GVHD and those without GVHD before (T1, pre-conditioning) and after the conditioning regimen (T2, pre-transplant). CEC values neither changed in relation to age and sex at any timepoints (data not shown). At T1 (pre-conditioning) MDS/CMS patients had lower CEC values in comparison to acute leukemia (p = 0.0001) and Lymphomas/CLL patients (p = 0.001) (Fig. 2A). At T2 (pre-transplant) no differences in CEC values were recorded depending on diagnosis and conditioning regimen (MAC vs RIC), while patients receiving TBI-based conditioning regimen showed higher CEC values (p = 0.02) (Fig. 2C). At T3 (engraftment) no differences in CEC values were recorded depending on diagnosis, conditioning regimen, donor type and cells source (data not shown), while CyA/MTX GVHD prophylaxis was associated with higher CEC values (p = 0.03) (Fig. 2E). CEC values at T3 resulted higher in patients without GVHD in comparison to patients developing GVHD (p = 0.01) (Fig. 3A). This difference remained significant in multivariate analysis by logistic regression model (OR 0.98, 95% C.I. 0.97–0.99; p = 0.02). At T4 (day +28 or GVHD onset) patients with GVHD had higher CEC values in comparison to patients without GVHD (p = 0.02) (Fig. 3A). This difference remained significant in multivariate analysis by logistic regression model (OR 0.98, 95% C.I. 0.97–0.99; p = 0.01). At T5 (1 week after steroids) CEC values returned to pre-transplant counts (data not shown).

### CEC counting with CellSearch

In CellSearch generated CEC values, no differences were detected between patients developing GVHD and those without GVHD at any timepoints. CEC values neither changed in relation to age and sex at any timepoints (data not shown). At T1 (pre-conditioning) AL patients had higher CEC values in comparison to MDS/MCS (p < 0.05) and Lymphomas/CLL patients (p < 0.05) (Fig. 2A). At T2 (pre-transplant) no differences in CEC values were recorded depending on conditioning regimen (MAC vs RIC), while patients receiving TBI-based conditioning regimen showed higher CEC values (p = 0.01) (Fig. 2C). At T3 (engraftment) no differences in CEC values were recorded depending on conditioning regimen, donor type, cells source and GVHD prophylaxis (data not shown). CEC values at T3 were relatively higher in patients without GVHD in comparison to patients developing GVHD (p = NS) (Fig. 3B). At T4 (GVHD onset or day +28) patients with GVHD had only few more CEC in comparison to patients without GVHD (p = NS) (Fig. 3B). At T5 (1 week after steroids) CEC values returned to pre-transplant counts (data not shown).

### Comparison, correlation and degree of agreement between PFC and CellSearch

**Figure 4:**
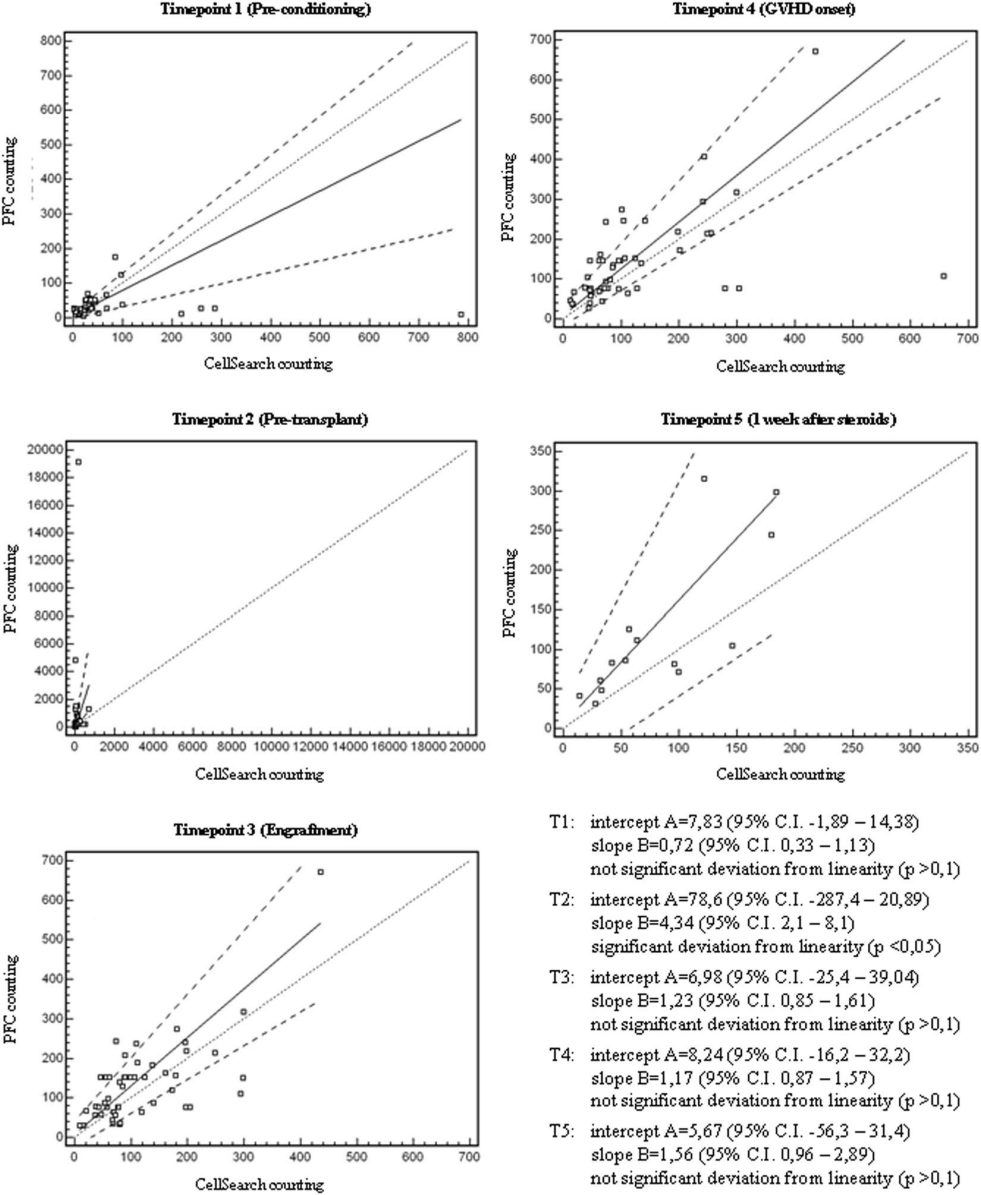
Linear correlation of CEC counts performed with CellSearch system and BD Lyotube (Custom cat # 623920). Linear correlation (Passing Bablok regression analysis) of CEC counts performed with CellSearch versus PFC (Lyotubes; BD Biosciences) at the different timepoints. Black line shows the regression line, dashed line the 95% C.I. of regression line, while dotted line represents the identity line (x = y).

**Figure 5:**
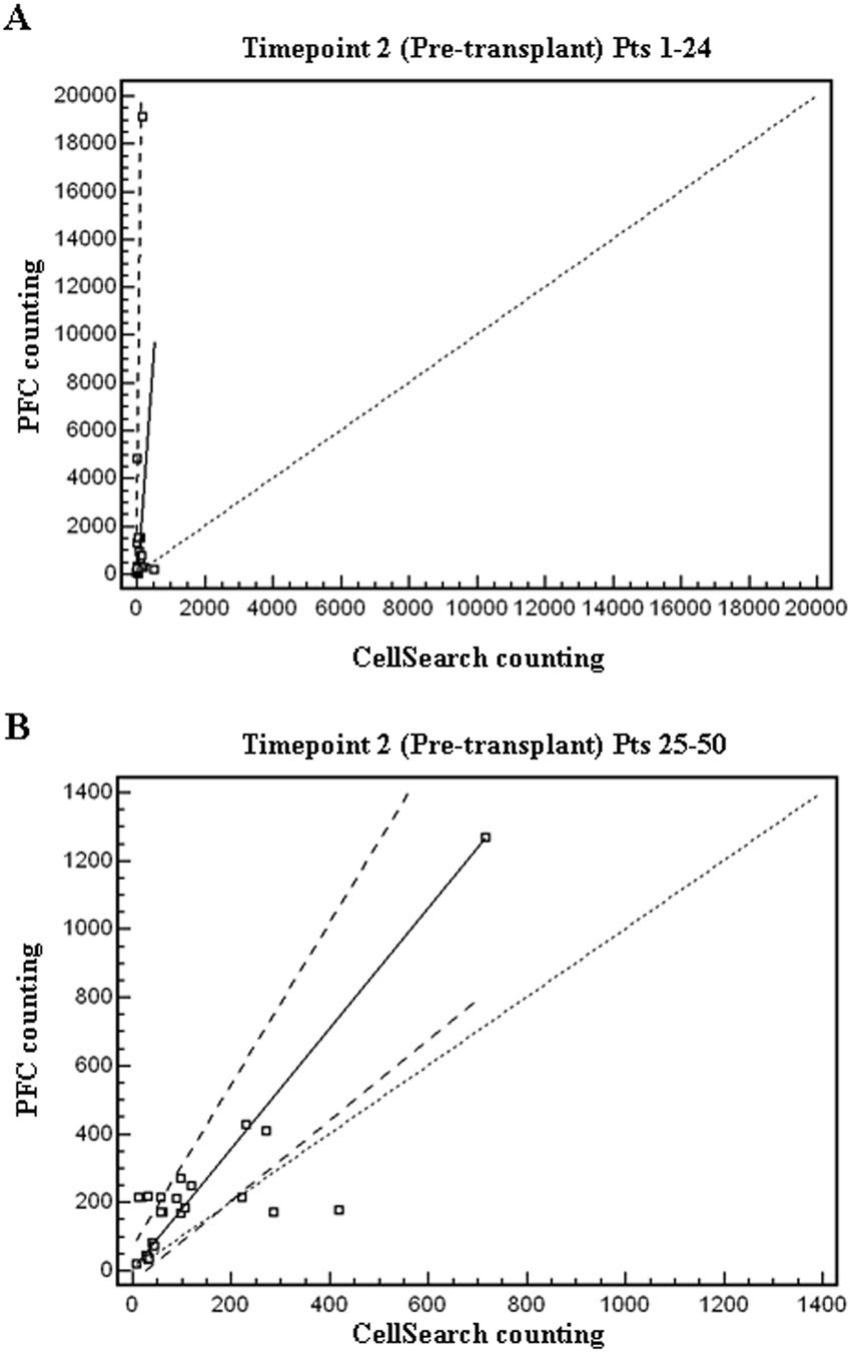
Linear correlation of CEC counts performed with CellSearch system and BD Lyotube (Custom cat # 623920) at T2 timepoint. Panel A shows the absence of correlation in the first 24 patients, in which the unreliability of lymphocytes absolute numbers obtained by standard cell counter in a very deep leukopenia phase, heavily affected the CEC counts determined by PFC. Intercept A = −905,7(−9816,8 to −112,6); slope B = 20,2 (6,5 to 155,6). Panel B shows the linear correlation from the 25^th^ patient onwards, in which lymphocytes absolute numbers was PFC determined (CD45^pos^ events in lympho-monocyte gate). Intercept A = 5,4 (−34,1 to 71,9); slope B = 1,8(1,2–2,37); not significant deviation from linearity (p > 0,05). Black line shows the regression line, dashed line the 95% C.I. of regression line, while dotted line represents the identity line (x = y).

In the present study, we have compared CEC counts in allo-HSCT patients by means of two different methodologies (PFC Lyotube versus CellSearch). Spearman rank correlation analysis showed a significant association between the CEC values of the two methodologies at all time points (Table 2). The effective comparability between PFC Lyotube and CellSearch was assessed by Bland Altman plot analysis and Passing Bablok regression analysis. Bias and limits of agreement between the two methods of CEC count (Bland Altman plot analysis) at each time point are shown in Table 2. The bias between PFC Lyotube and CellSearch resulted not significant at T1, T3, T4. At T2 and T5, the bias was not estimable, since the differences between the 2 methods were not normally distributed even after a logarithmic transformation of the data. Passing Bablok regression analysis showed not significant differences between CellSearch and PFC Lyotube except at T2 (Fig. 4). At T2 a significant deviation from linearity (p < 0.05) was observed, hence indicating that the Passing Bablok method is not applicable. However, when from the 25^th^ patient onwards lymphocytes’ absolute numbers at T2 was PFC determined (CD45^pos^ events in lympho-monocyte gate), no significant deviation from linearity can be recorded in the two splitted groups (patients 1–24 versus patients 25–50); moreover the regression line of CEC counts in patients from 25^th^ to 50^th^ showed a high comparability between CellSearch and PFC (Fig. 5). We thus confirmed that the lack was mainly related to the dual platform calculation procedure for PFC counts, being CEC determinations heavily affected at T2 by the unreliability of lymphocytes’ absolute numbers obtained by standard cell counter in a very deep leukopenia phase. Despite of the significant correlation and the satisfactory degree of agreement between PFC Lyotube and CellSearch, the two CEC counting procedures maintain a fairly series of pros and cons, that makes them not mutually exclusive, but rather complementary (Table 3). The weaknesses of one CEC counting procedure are strengths of the other one and *viceversa*.

**Table 2:** Comparison, correlation and degree of agreement between flowcytometry Lyotube and CellSearch for CEC count (n = 50 patients).

Time points	CEC/ml		p
	PFC Lyotube	CellSearch	
T1 (pre-conditioning) Median (range) Mean ± SD	24 (3–175) 33.4 ± 32.9	24 (2–786) 59.7 ± 122.7	0.63
-Spearman rank correlation -Bland Altman plot analysis	-r = 0.63; 95% C.I. (0.37–0.79) -limits of agreement (range)*: from −286.3 to 226.2 mean difference (d^) (95% C.I.) = bias: −30.1 (−68.7–9.7)		<0.0001 bias not significant^†^
T2 (pre-transplant) Median (range) Mean ± SD	214.5 (16–19123) 1046 ± 3313.1	64 (8–718) 114.3 ± 139	0.0009
-Spearman rank correlation -Bland Altman plot analysis	-r = 0.63; 95% C.I. (0.32–0.80) -limits of agreement (range)*: from −4705.9 to 6029.2 mean difference (d^) (95% C.I.) = bias: 661.6 (−116.7–1439.9)		0.0001 significance of bias not estimable
T3 (engraftment) Median (range) Mean ± SD	124 (30–670) 145.5 ± 122.4	85 (10–436) 120.2 ± 89.6	0.43
-Spearman rank correlation -Bland Altman plot analysis	-r = 0.79; 95% C.I. (0.61–0.88) -limits of agreement (range)*: from −129.5 to 166.3 mean difference (d^) (95% C.I.) = bias: 18.4 (−3–39.8)		<0.0001 bias not significant^†^
T4 (GVHD onset) Median (range) Mean ± SD	104.5 (26–670) 140.5 ± 112.6	83 (13–658) 127.5 ± 122.9	0.38
-Spearman rank correlation -Bland Altman plot analysis	-r = 0.58; 95% C.I. (0.34–0.74) -limits of agreement (range)*: from −204.9 to 238.3 mean difference (d^) (95% C.I.) = bias: 16.7 (−15.4–48.8)		0.0001 bias not significant^†^
T5 (1 week after steroids) Median (range) Mean ± SD	104.5 (26–670) 121.3 ± 93.9	64 (14–184 85.3 ± 54.4	0.27
-Spearman rank correlation -Bland Altman plot analysis	-r = 0.79; 95% C.I. (0.43–0.92) -limits of agreement (range)*: from −79.1 to 157.1 mean difference (d) (95% C.I.) = bias: 39 (4.2–73.8)		0.0008 significance of bias not estimable

^*^Limits of agreement = range within which 95% of the differences between the measurements of the two methods are included.

d^ = mean of the differences between the paired measurements of the two methods.

^†^The bias is not significant if the 95% confidence interval of the mean difference includes the value 0 (line of equality on the Bland Altman plot); in the BA analysis, differences between two methods (y) are plotted vs. the means of the two measurements (x); the bias (mean difference) is represented by the gap between the X axis, corresponding to zero differences between the 2 methods (line of equality), and the parallel line to the X axis at the value of the bias.

**Table 3:** Pros and cons of the two different CEC counting procedures.

	Pros	Cons
CellSearch	Analysis within 96 hrs Sample preparation/staining fully automated Single platform count	Only one channel free for additional MoAb Occasionally high number of images (>10000) CEC counting semi-automated Need of specific training Highly expensive
PFC Lyotube	Highly performing Widespread platform availability Implementable (from 6-color standard)	Sample staining/acquisition within 6 hrs Sample preparation/staining manually done Double platform count Highly trained personnel with expertise Time consuming for acquisition (4 M events)

## Discussion

CEC are rare cellular subpopulations in PB, detaching from vessel walls as a consequence of vascular damage or their physiological turnover. Together with endothelial progenitor cells (EPC) provide a measure of vascular health balance, being CEC markers of ongoing endothelial damage, whereas EPC informative of endothelial repair suitability^9,11,12,24–28^. In several clinical conditions, CEC counts have been exploited as valuable biomarkers to monitor vascular damage and treatment response^9–14^. However, due to their rareness and complex immunophenotype, no consensus has yet been fully reached on their identification and way of count^15–17^. CEC count changes, as function of endothelial damage, have been reported in many different clinical conditions^9–14^. However, the translation of CEC counts into clinical practice has been weakened by the inconsistent results available till now. Recently, we joined the S.C.EN.I.C. network that developed a highly optimized PFC protocol for accurate CEC identification and count^18,19^, defining CEC as live and nucleated CD34^bright^/CD45^neg^/CD146^pos^ events. Moreover, a physiological baseline range for healthy subjects has been accounted, suitable as starting point for CEC monitoring in endothelial dysfunctions^19^. Reliability and reproducibility of S.C.EN.I.C. results have been consequently confirmed in a typical endothelial damage disease (i.e. diabetes)^24^ and in coronary artery disease patients (REMEDY clinical trial)^25^. Furthermore, by using the CellSearch system, we have just recently confirmed the role of CEC changes in the diagnostic definition of GVHD in the largest series of allo-HSCT patients^20^. Based on these assumptions, the aims of the present study were to (i) test an *ad hoc* designed PFC-based panel for CEC counting in the allo-HSCT setting; (ii) evaluate the agreement of the PFC-based panel with the validated CellSearch system; (iii) confirm that CEC changes in allo-HSCT patients represents a suitable tool to support clinicians in the GVHD diagnosis. The innovation of our study derives from the use of the validated and commercially available CellSearch system in comparison to an *“ad hoc”* designed novel polychromatic protocol using a lyophilized antibody panel (BD Lyotube, Custom cat #623920), that proved to be the most reliable and suitable PFC method to count CEC, recently available on the market^19^. Even though clear phenotypic CEC definition has not yet reached widespread consensus, reasonable agreement has been proved between PFC and immunomagnetic-based methods for CEC quantification in whole blood^15,29^. All together both methodologies still show a few issues; benefits and drawbacks have been in depth analysed by Goon *et al*.^15^, pointing out a caution against using both techniques interchangeably. By comparing CEC values (PFC Lyotube versus CellSearch) in allo-HSCT patients at the five different timepoints, we have highlighted a significant comparability at all timepoints, except for the pre-transplant one (T2). This lack was mainly related to the dual platform calculation procedure for PFC counts, being CEC determinations heavily affected at T2 by the unreliability of lymphocytes absolute numbers obtained by standard cell counter in a very deep leukopenia phase. Thus, from the 25^th^ patient onwards, lymphocytes absolute numbers at T2 was PFC determined using a dedicated TruCount tube (BD) containing CD45 and absolute counting beads (CD45^pos^ events in lympho-monocyte gate). This modification allowed us to obtain a more satisfactory correlation between the two counting methods. It could be argued that a step forward in PFC counting improvement could be done by switching to a single tube assay, performing CEC identification and absolute count in the same step; however this approach would be limited by technical concerns related to the very high number of events to be acquired, fluorescences compliance and the essential washing step required by the PFC procedure. By extending in wider details what reported in Table 3, CellSearch system has the great advantage, by using dedicated tubes (CellSave Preservative Tubes), to allow CEC counting within 96 hours from PB drawing, making it possible to easily pull together samples from different days or to send them to a centralized lab-facility. Since CellSearch system, after on-board samples loading, operate in a fully automated manner, personnel can be used for different tasks during the three hours running time. Last but not least, CellSearch system provides results expressed in number of CEC per mL of PB without further calculations needed. The aforementioned aspects are anyhow counterbalanced by a few unfavourable factors. CellSearch system, besides APC and PE fluorescence channels dedicated to anti-CD45 and anti-CD105, has only one more channel available, dedicated to FITC fluorescence detection. Therefore, availability of fluorescence combinations represents a fairly cumbersome limitation, particularly when dealing with phenotypically complex cellular events to identify. Moreover, since CellSearch system software operates semi-automatically, capturing all images showing fluorescence signals in the PE channel, sometimes several thousands of images are presented to the operator for CEC identification and consequent count. Furthermore, personnel need to be specifically trained. Finally, it should not be forgotten the high costs for equipment purchase.

On the other side, lights and shadows are present also for the PFC procedure. First of all, PFC gives the valuable advantage to study additional antigens within the assay format (i.e. VEGFR2, for EPC identification and count) or easily to implement from the standard 6-color configuration up to combinations of 8–10 fluorescences, coupled with the prevention of CEC underestimation due to cell loss, because an immunological pre-enrichment step is avoided^29^. The widespread diffusion of PFC instruments makes accessibility to technology easy and immediate. The dark sides include the need to perform counting shortly after PB drawing, to avoid unpredictable decrease in CEC^19^ and EPC^23^ values overtime. Furthermore, cryopreservation exerts detrimental effects making CEC counting unreliable on thawed samples^19^. Highly trained personnel with expertise is required due to the rareness and complexity of cellular populations to be recognized and clearly identified; sample preparation and staining is done manually and sample acquisition and analysis (4 million events per sample), requiring accuracy and precision, is time consuming. By the end, the double platform count is heavily affected by deep leukopenia phase that, however, can be nicely solved by PFC determination of lymphocytes absolute numbers (CD45^pos^ events in lympho-monocyte gate).

The present study represents the only comparison trial, performed to date, that confirms the potential role of CEC count changes as a suitable tool to support clinicians in GVHD diagnosis. Our results strengthen the linear correlation between either methods, but furthermore points out the intrinsic limitations of each one procedure. Indeed, despite both techniques are considered suitable for CEC counting, they utilise fairly different analytical paths to provide results. Therefore, by taking into account the economic resources to be employed, we would recommend either one procedure for supporting GVHD diagnosis in locally-based routine application, but in case of multicenter trial in more exploratory setting (i.e. chronic GVHD, veno-occlusive disease, idiopathic pneumonia syndrome) the use of both techniques could be advised.

Although allo-HSCT is considered a potential curative therapy for patients affected by hematological disorders (i.e. malignancies, hematological deficiencies and immune disorders) and has improved the survival expectations of many patients, it is still not without risks, being GVHD the most life-threatening complication^2–4^. To date, no laboratory test can predict, on a routine basis, the risk of developing GVHD or the responsiveness to treatment. The availability of validated GVHD biomarkers, from PB samples, could result in an earlier identification of patients burdened by higher risk of GVHD manifestations^30,31^. Consequently, patient identification will enable risk-adapted approaches to GVHD treatment, primarily implying an early and motivated switch to additional immunosuppressive therapies before the development of treatment unresponsiveness or refractoriness. Moreover, the endothelial damage occurring during allo-HSCT might represent an emerging and intriguing target for future studies involving preventive approaches^32^ or monitoring angiogenesis-inhibiting therapies. However, further studies on much larger patient numbers need to be performed in order to provide reliable and unquestionable answers to those issues.

## Electronic supplementary material

Supplemental Figure

## Data Availability

The datasets generated and analyzed during the current study are available from the corresponding author on reasonable request.

## References

[1] Appelbaum FR (2007). Hematopoietic-Cell Transplantation at 50. N Engl J Med.

[2] Pidala J (2011). Graft-vs-host disease following allogeneic hematopoietic cell transplantation. Cancer Control.

[3] Blazar BR, Murphy WJ, Abedi M (2012). Advances in graft-versus-host disease biology and therapy. Nature Reviews Immunology.

[4] Jagasia M (2012). Risk factors for acute GVHD and survival after hematopoietic cell transplantation. Blood.

[5] Dignan FL (2012). Diagnosis and management of acute graft-versus-host disease. Br J Haematol.

[6] Chen Y-B, Cutler CS (2013). Biomarkers for acute GVHD: can we predict the unpredictable?. Bone Marrow Transplant.

[7] Biedermann BC (2002). Endothelial injury mediated by cytotoxic T lymphocytes and loss of microvessels in chronic graft versus host disease. Lancet.

[8] Carreras E, Diaz-Ricart M (2011). The role of the endothelium in the short-term complications of hematopoietic SCT. Bone Marrow Transplant.

[9] Dignat-George F, Sampol J (2000). Circulating endothelial cells in vascular disorders: new insights into an old concept. Eur J Haematol.

[10] Rowand JL (2007). Endothelial cells in peripheral blood of healthy subjects and patients with metastatic carcinomas. Cytometry A.

[11] Erdbruegger U, Dhaygude A, Haubitz M, Woywodt A (2010). Circulating Endothelial Cells: Markers and Mediators of Vascular Damage. Curr Stem Cell Research & Therapy.

[12] Fadini GP, Losordo D, Dimmeler S (2012). Critical reevaluation of endothelial progenitor cell phenotypes for therapeutic and diagnostic use. Circ Res.

[13] Damani S (2012). Characterization of circulating endothelial cells in acute myocardial infarction. Sci Transl Med.

[14] Almici C (2014). Changes in Circulating Endothelial Cells Count Could Become a Valuable Tool in the Diagnostic Definition of Acute Graft-Versus-Host Disease. Transplantation.

[15] Goon PKY, Boos CJ, Stonelake PS, Blann AD, Lip GYH (2006). Detection and quantification of mature circulating endothelial cells using flowcytometry and immunomagnetic beads: a methodological comparison. Thromb Haemost.

[16] Widemann A (2008). CD146-based immunomagnetic enrichment followed by multiparameter flow cytometry: a new approach to counting circulating endothelial cells. J Thromb Haemost.

[17] Mancuso P (2009). Validation of a standardized method for enumerating circulating endothelial cells and progenitors: Flow cytometry and molecular and ultrastructural analyses. Clin Cancer Res.

[18] Lanuti P (2016). Endothelial Progenitor Cells, Defined by the Simultaneous Surface Expression of VEGFR2 and CD133, are not Detectable in Healthy Peripheral and Cord Blood. Cytometry A.

[19] Lanuti P (2018). A standardized flow cytometry network study for the assessment of circulating endothelial cell physiological ranges. Scientific Reports.

[20] Almici C (2017). Circulating endothelial cells count: a reliable marker of endothelial damage in patients undergoing hematopoietic stem cell transplantation. Bone Marrow Transplant.

[21] De Pauw B (2008). Revised definitions of invasive fungal disease from the European Organization for Research and Treatment of Cancer/Invasive Fungal Infections Cooperative Group and the National Institute of Allergy and Infectious Diseases Mycoses Study Group (EORTC/MSG) Consensus Group. Clin Infect Disease.

[22] Przepiorka (1995). 1994 Consensus Conference on acute GVHD grading. Bone Marrow Transplant.

[23] Kharabi Masouleh B, Baraniskin A, Schmiegel W, Schroers R (2010). Quantification of circulating endothelial progenitor cells in human peripheral blood: Establishing a reliable flow cytometry protocol. J Immunol Methods.

[24] Cappellari R, D’Anna M, Avogaro A, Fadini GP (2016). Plerixafor improves the endothelial health balance. The effect of diabetes analysed by polychromatic flow cytometry. Atherosclerosis.

[25] Madonna R (2017). The acute impact of high-dose lipid-lowering treatment on endothelial progenitor cells in patients with coronary artery disease - The REMEDY-EPC early substudy. PLoS One.

[26] Hill JM (2003). Circulating endothelial progenitor cells, vascular function and cardiovascular risk. N Engl J Med.

[27] Asahara T (1997). Isolation of putative progenitor endothelial cells for angiogenesis. Science.

[28] Fadini GP, Avogaro A (2010). Cell-based methods for *ex vivo* evaluation of human endothelial biology. Cardiovasc Res.

[29] Kraan J (2012). A new approach for rapid and reliable enumeration of circulating endothelial cells in patients. J Thromb Haemost.

[30] Paczesny S (2013). Discovery and validation of graft-versus-host disease biomarkers. Blood.

[31] Holtan SG, MacMillan ML (2016). A risk adapted approach to acute GVHD treatment: are we there yet?. Bone Marrow Transplant.

[32] Mir E (2017). Endothelial damage is aggravated in acute GvHD and could predict its development. Bone Marrow Transplant.

