# Supplementary material for: Counting circulating endothelial cells in allo-HSCT: an ad hoc designed polychromatic flowcytometry-based panel versus the CellSearch System
Source: Sci Rep. 2019 Jan 14;9:87. doi: 10.1038/s41598-018-36442-9 (PMC6331628; doi:10.1038/s41598-018-36442-9)
Supplement: Supplementary file 1 — Supplemental Figure [file 41598_2018_36442_MOESM1_ESM.doc]

Counting circulating endothelial cells in allo-HSCT: an *ad hoc* designed polychromatic flowcytometry-based panel versus the CellSearch System.

*Camillo ALMICI^1^, Arabella NEVA^1^, Cristina SKERT^2^, Benedetto BRUNO^3^, Rosanna VERARDI^1^, Andrea DI PALMA^2^, Andrea BIANCHETTI^1^, Simona BRAGA^1^, Giovanna PIOVANI^4^, Valeria CANCELLI^2^, Paola OMEDE’^3^, Kurt BAETEN^5^, Gianluca ROTTA^6^, Domenico RUSSO^2^ and Mirella MARINI^1^

^1^Laboratory for Stem Cells Manipulation and Cryopreservation, Dpt of Transfusion Medicine, ASST Spedali Civili, Brescia, Italy

^2^Chair of Hematology, Unit of Blood Diseases and Stem Cell Transplantation, University of Brescia, ASST Spedali Civili, Brescia, Italy

^3^BMT Unit, Department of Molecular Biotechnology and Health Sciences, University of Torino, A.O.U Città della Salute e della Scienza di Torino, Torino, Italy

^4^Biology and Genetics Division, Department of Molecular and Translational Medicine, University of Brescia, Brescia, Italy

^5^Global Scientific and Medical Affairs, Janssen Diagnostics, Beerse, Belgium

^6^Scientific Affairs, BD Biosciences Italia, Milano, Italy.

Corresponding Author: Dr. Camillo ALMICI, Laboratory for Stem Cells Manipulation and Cryopreservation, Department of Transfusion Medicine - ASST Spedali Civili di Brescia, Pz.le Spedali Civili, 1 – 25123 Brescia, Italy; Phone: Italy+030 3996579, Fax: Italy+030 3996066

[camillo.almici@asst-spedalicivili.it](mailto:camillo.almici@asst-spedalicivili.it)

**Suppl Table S1. Comparison of clinical and transplant characteristics in patients with and without acute GvHD.**

| **Characteristics** | **GvHD (%)** | **No GvHD (%)** | **p** |
| --- | --- | --- | --- |
| Age (years), median (range) | 47.5 (25-67) | 54.5 (18-69) | 0.60 |
| Sex  Male  Female | 10 (50)  10 (50) | 20 (67)  10 (33) | 0.24 |
| Diagnosis  Acute Leukemias  Lymphomas/CLL  MDS  CMS | 17 (85)  2 (10)  0 (0)  1 (5) | 17 (57)  6 (20)  5 (17)  2 (6) | **0.03**  0.34  0.05  0.81 |
| Disease status  CR  PR/CR>1  Progression | 14 (70)  6 (30)  0 (0) | 15 (50)  13 (43)  2 (7) | 0.16  0.34  0.09 |
| Donor  MUD  MRD  Haploid | 9 (45)  1 (5)  10 (50) | 17 (57)  7 (23)  6 (20) | 0.42  0.08  **0.02** |
| HPC source  MPB  BM | 15 (75)  5 (25) | 24 (80)  6 (20) | 0.68 |
| Conditioning regimen  MAC  RIC  TBI (yes) | 14 (70)  6 (30)  3 (15) | 16 (53)  14 (47)  6 (20) | 0.24  0.65 |
| MA conditioning  BU/CY  FBu4  TBI/CY  TBI/F/Th  TBF  RIC conditioning  Th/CY/F  TBI/F/Th(r)  FBu2  Th/CY  TBF(r)  Th/F | 4 (20)  3 (15)  1 (5)  2 (10)  4 (20)  1 (5)  0 (0)  0 (0)  2 (10)  3 (15)  0 (0) | 7 (23)  1 (3)  3 (10)  1 (3)  4 (14)  1 (3)  2 (8)  6 (20)  3 (10)  1 (3)  1 (3) | 0.78  0.14  0.52  0.33  0.53  0.77  0.24  **0.03**  1.00  0.14  0.41 |
| GVHD prophylaxis *  CyA/MTX  CyA/MMF  ATG | 10 (50)  10 (50)  6 (30) | 23 (77)  7 (23)  13 (43) | 0.05  0.34 |

CLL: Chronic Lymphocitic Leukaemia, MDS: Myelodiplastic Syndrome, CMS: Chronic Myeloproliferative Syndromes, MUD: Matched Unrelated Donor, MRD: Matched Related Donor, Haploid: haplodentical related donor, MPB: Mobilised Peripheral Blood, BM: Bone Marrow, MAC: myeloablative conditinong, RIC: reduced intensity conditioning, BU: Busulphan, CY: Cyclophosphamide, F: Fludarabine, TBI: Total Body Irradiation, Th: Thiotepa, TBF: Th/BU/F, r: RIC, MTX: Methotrexate, MMF: Mofetil Micofenolate, ATG: Anti-Lymphocyte Globulin.

*in haploidentical transplantation GVHD prophylaxis included CyA/MMF and Cyclophosphamide post stem cell reinfusion (100 mg/kg total dose: day+3 and +5).

**Suppl Table S2. PFC raw data for calculating CEC values by applying the indicated formula (18).**

| **Timepoint** | **# CEC**  **panel** | **# CEC**  **control** | **# Total CD34**  **panel** | **# Total CD34**  **control** | **# Lymphocyte**  **panel** | **Lymphocyte**  **count** | **CEC/ml** |
| --- | --- | --- | --- | --- | --- | --- | --- |
| T1 (preconditioning) | 13 | 2 | 17099 | 17211 | 1349826 | 870 | 7 |
| T2 (pretransplant) | 232 | 3 | 307 | 345 | 1793 | 10 | 1279 |
| T3 (engraftment) | 178 | 11 | 1280 | 965 | 882586 | 590 | 109 |
| T4 (GVHD onset) | 348 | 0 | 1607 | 1782 | 2416221 | 1490 | 215 |
| T5 (1week after steroids) | 98 | 1 | 446 | 445 | 362272 | 390 | 104 |

$$CEC/mL=\frac{\#CEC_{panel}- \#CEC_{control}\times\#Total CD34_{panel}/ \#Total CD34_{control}}{\#Lymphocyte_{panel}} \times Lymphocyte Count\times1000$$

#CEC_panel_ events counted as CEC (CD34bright/CD45neg/CD146pos), after running the tube containing the whole panel of reagents.

#CEC_control_ events in the CEC gate, after running the control tube, containing the CD146 isotype control and all the remaining surface reagents present in the panel.

#Total CD34_panel_ total CD34pos events (number of HSC + number of CD34bright/CD45neg cells), after running the tube containing the whole panel of reagents.

#Total CD34_control_ total CD34pos events (number of HSC + number of CD34bright/CD45neg cells), after running the control tube.

#Lymphocyte_panel_ lymphocytes gated on the CD45/SSC dot plot after running the panel tube.

Lymphocyte Count absolute number of lymphocytes obtained from a standard cell counter.


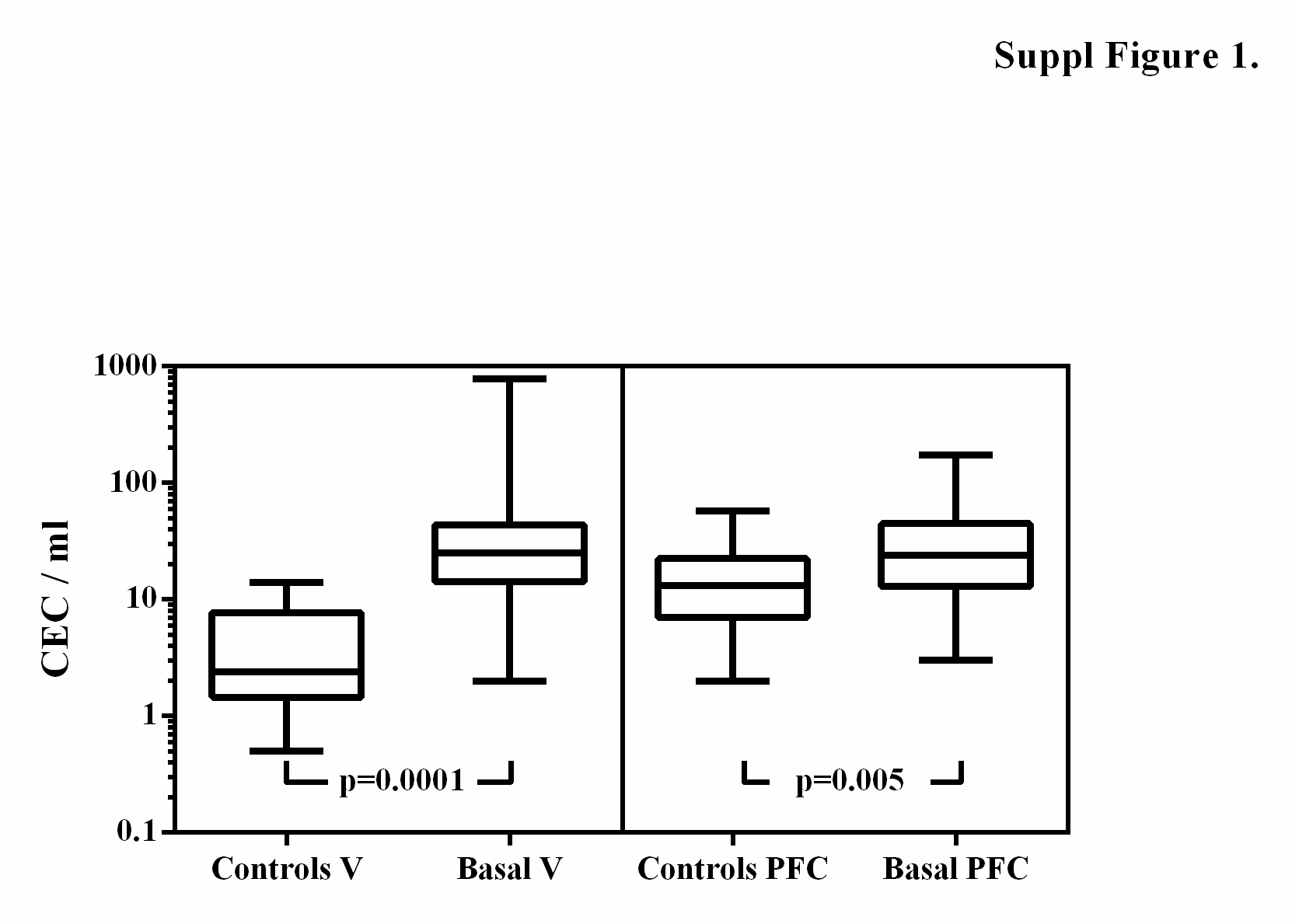


**Suppl Figure S1. CEC counts at baseline (T1, pre-conditioning) in patients undergoing allo-HSCT in comparison to healthy subjects (controls).**

On the left section of the graph CEC counts performed with CellSearch (V) and on the right part with polychromatic flow cytometry (PFC) (controls V: n=17; Basal V: n=48; controls PFC: n=21; Basal PFC: n=39). Boxes represents values from the first to the third quartile, horizontal line shows the median value and the whiskers indicate the min and max value.
